# GenBank is a reliable resource for 21st century biodiversity research

**DOI:** 10.1073/pnas.1911714116

**Published:** 2019-10-21

**Authors:** Matthieu Leray, Nancy Knowlton, Shian-Lei Ho, Bryan N. Nguyen, Ryuji J. Machida

**Affiliations:** ^a^Smithsonian Tropical Research Institute, Smithsonian Institution, Panama City, 0843-03092, Republic of Panama;; ^b^National Museum of Natural History, Smithsonian Institution, Washington, DC 20560;; ^c^Biodiversity Research Centre, Academia Sinica, 115-29 Taipei, Taiwan;; ^d^Department of Biological Sciences, The George Washington University, Washington, DC 20052;; ^e^Computational Biology Institute, Milken Institute School of Public Health, The George Washington University, Washington, DC 20052

**Keywords:** environmental DNA, metabarcoding, taxonomic assignments

## Abstract

Traditional methods of characterizing biodiversity are increasingly being supplemented and replaced by approaches based on DNA sequencing alone. These approaches commonly involve extraction and high-throughput sequencing of bulk samples from biologically complex communities or samples of environmental DNA (eDNA). In such cases, vouchers for individual organisms are rarely obtained, often unidentifiable, or unavailable. Thus, identifying these sequences typically relies on comparisons with sequences from genetic databases, particularly GenBank. While concerns have been raised about biases and inaccuracies in laboratory and analytical methods, comparatively little attention has been paid to the taxonomic reliability of GenBank itself. Here we analyze the metazoan mitochondrial sequences of GenBank using a combination of distance-based clustering and phylogenetic analysis. Because of their comparatively rapid evolutionary rates and consequent high taxonomic resolution, mitochondrial sequences represent an invaluable resource for the detection of the many small and often undescribed organisms that represent the bulk of animal diversity. We show that metazoan identifications in GenBank are surprisingly accurate, even at low taxonomic levels (likely <1% error rate at the genus level). This stands in contrast to previously voiced concerns based on limited analyses of particular groups and the fact that individual researchers currently submit annotated sequences to GenBank without significant external taxonomic validation. Our encouraging results suggest that the rapid uptake of DNA-based approaches is supported by a bioinformatic infrastructure capable of assessing both the losses to biodiversity caused by global change and the effectiveness of conservation efforts aimed at slowing or reversing these losses.

## Significance

As loss of biodiversity and ecosystem degradation become major concerns worldwide, scientists increasingly depend on DNA-based characterization of animal communities for monitoring and impact assessments. These analyses ultimately depend on the taxonomic reliability of genetic databases for taxonomic assignments. Concerns have been raised about the reliability of GenBank, the largest and most widely used genetic database. We show that, contrary to expectations, the proportion of mislabeled sequences in GenBank is surprisingly low. Major taxonomic errors are vanishingly small (0.01% at the class level, 0.05% at the order level), and likely <1% even at the genus level. These results show that GenBank is much more reliable for a range of applications, including studies of environmental change, than previously thought.

Human activities have resulted in deteriorating environmental conditions worldwide, prompting urgent calls to increase our ability to assess biodiversity across space and through time (1, 2). Identifying organisms for these assessments typically relies on observations or, for small organisms, reference to voucher specimens housed in collections. Traditional morphological identifications of collected materials are increasingly being replaced by DNA-based identifications. This change has been accelerated by the adoption of DNA barcoding (3) because of its accuracy, speed, and now lower costs. However, for very small and abundant organisms (the vast majority of diversity on the planet), even traditional barcoding can be impractical. Thus, sequencing of bulk DNA samples (from collections of organisms) or environmental DNA (eDNA; DNA shed from organisms into the environment) via metabarcoding (the characterization of multiple DNA sequences from a single sample) is increasingly being used to characterize biodiversity in terrestrial, freshwater, and marine habitats (4, 5). Materials studied include bulk soil (6), sediment, and benthic aquatic samples (7); bulk samples from organismal traps (e.g., Malaise traps for insects) (8); gut contents (9); plankton tows (9); filtered water (10); and ancient DNA (11, 12).

Identifying animal (metazoan) sequences obtained from these environmental samples typically relies on comparisons with GenBank (13), the largest repository of genetic data for biodiversity (14, 15). In many cases, no vouchers are available to independently confirm identification, because the organisms are tiny, very difficult or impossible to identify, or lacking entirely (in the case of eDNA). While concerns have been raised about biases and inaccuracies in laboratory and analytical methods used in metabarcoding (16–18), comparatively little attention has been paid to the taxonomic reliability of GenBank itself, whose >4.7 million mitochondrial gene sequences representing >170,000 metazoan species have never been comprehensively assessed for taxonomic accuracy.

Mitochondrial sequences represent an invaluable resource because of their comparatively rapid evolutionary rates and consequent high taxonomic resolution (19). The majority of metazoan mitochondrial genomes contain 37 genes, including 2 coding for ribosomal RNA, 13 coding for proteins, and 22 tRNAs (20). The tRNAs generally are very short (approximately 60 to 70 bp) and thus not useful as taxonomic markers. However, the other 15 genes are generally much longer than 100 bp, making them potentially useful markers. For the many small and often undescribed organisms that represent the bulk of animal diversity (21), they represent a cost-effective way of broadly characterizing individuals, populations, species, and communities.

Incorrect taxonomic annotations of DNA sequence data can be caused by inadvertent amplification of laboratory contaminants (22), intimately associated organisms including bacteria (23, 24), or nontarget genes, such as pseudogenes (25); by incorrect identification of the study organisms (26); or by mistakes made during data entry at various stages. These annotation errors could be pervasive and highly problematic, but there have been only limited attempts to quantify their magnitude. We analyzed the scale, patterns, and causes of metazoan mitochondrial sequence mislabeling in the GenBank BLAST NT database at the genus level and above to address previous critiques of GenBank that have focused on major taxonomic errors. We used VSEARCH (27) to group closely related metazoan sequences of 13 protein-coding and 2 ribosomal RNA-coding mitochondrial genes (*SI Appendix*, Table S1) at 97%, 98%, 99%, and 100% thresholds. Clusters containing sequences belonging to different phyla, classes, orders, families, or genera were flagged for further investigation. This approach assumes that clusters of highly similar sequences (97% or greater) for these hypervariable genes should typically contain conspecifics but not sequences of multiple genera, families, orders, classes, and phyla (unless they are mislabeled). We did not attempt to identify errors at the species level because to do so would require case-by-case assessments of far more clusters coupled with detailed taxonomic evaluations, since multispecies clusters could often be the result of insufficient taxonomic resolution of the genes or taxonomic disagreements and revisions.

## Results and Discussion

**Fig. 1.**
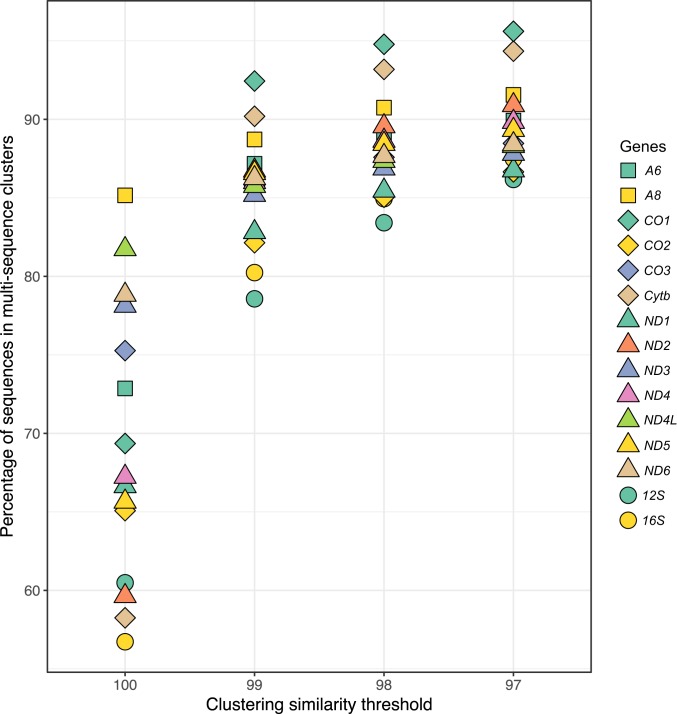
Percentage of sequences in multisequence clusters for 13 protein and 2 ribosomal RNA-coding metazoan mitochondrial encoded genes. Clustering was performed on sequences retrieved from the GenBank BLAST nucleotide database using VSEARCH.

A total of 4,714,864 gene sequences downloaded from GenBank yielded 279,899, 304,804, 354,463, and 440,800 multisequence clusters (groups containing more than 1 sequence) at 97%, 98%, 99% and 100% clustering thresholds, respectively (*SI Appendix*, Table S1 provides the breakdown per gene at the 97% threshold). The remaining 332,834, 385,985, 511,172, and 1,547,404 “clusters,” respectively, had just a solitary sequence, which by definition could not be used to test for labeling errors since the test depends on having more than 1 higher taxon in a cluster. As expected, sequences in multisequence clusters represented a larger proportion of all sequences at lower similarity thresholds (Fig. 1). Cytochrome oxidase subunit I (*CO1*) and Cytochrome *b* apoenzyme (*Cytb*), the 2 most common mitochondrial gene sequences in GenBank, with 54.5% and 10.5% of all mitochondrial sequences, respectively, also had somewhat higher proportions of sequences in multisequence clusters (e.g., >90% at a 97% threshold; Fig. 1 and *SI Appendix*, Fig. S1), probably because with a greater depth of sampling, sequences that do not form clusters are less likely. Thus, we highlight analyses based on the 97% threshold for these 2 genes, as these estimates are the most inclusive of GenBank’s data and hence likely to be the most reliable. We also present analyses of the small (*12S*) and large (*16S*) ribosomal RNA genes, as they are being increasingly targeted in community DNA studies that heavily rely on GenBank for taxonomic annotations (4). The 97% threshold represents a level of sequence similarity typical of intraspecific variation for mitochondrial genes (28); it also provides a reasonable upper bound on the estimate of mislabeled sequences because the resulting larger clusters are more likely to contain genuinely related higher taxa (e.g., congeners) that nevertheless will be flagged as misidentified by virtue of belonging to a single cluster.

To estimate the number of sequences that were incorrectly annotated, we examined all clusters containing multiple phyla, classes, and orders individually and used phylogenetic analyses to determine where the errors occurred. Whenever mislabeled sequences at the order, class, and phylum levels could not be identified unequivocally with phylogenetic analysis, we calculated the minimum and maximum possible number of misannotated sequences, assuming that at least 1 annotation was correct. For the much more numerous clusters containing multiple families or genera, we used the same methods as were used for higher taxonomic ranks for all the clusters with 100 or more sequences (i.e., 375 clusters accounting for 133,598 sequences); for clusters with fewer than 100 sequences, we only estimated the minimum and maximum numbers of mislabeled sequences. We manually examined clusters larger than 100 sequences because our analyses showed that checking these clusters (which represented only 2.0% of all clusters but contained 46.8% of the total sequences) was an efficient way of improving the precision of our estimate of the possible range of error rates (*SI Appendix*, Fig. S2).

**Fig. 2.**
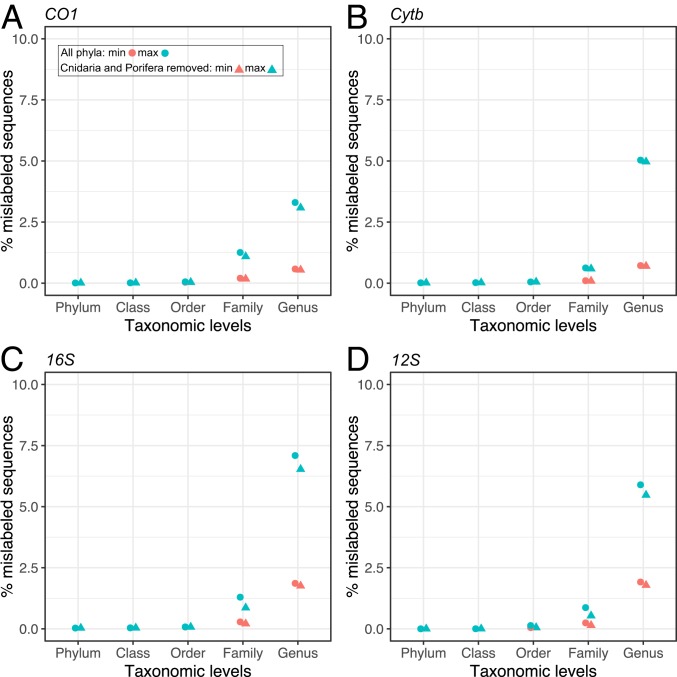
Estimated percentage of mislabeled metazoan sequences for 2 protein coding genes and 2 ribosomal RNA coding genes: (*A*) *CO1*; (*B*) *Cytb*; (*C*) *16S*; and (*D*) *12S*. Estimated minimum and maximum values are indicated for each taxonomic level. Calculations were made with and without the phyla Cnidaria and Porifera because they are known to have lower rates of evolution for these genes; however, these 2 groups account for only 0.6% and 0.09% of all sequences, respectively, and thus have a relatively minor influence on overall error estimates.

The percentage of sequences with incorrect assignments increased with decreasing taxonomic rank but was very low for most taxonomic levels (Table 1 and Fig. 2). Only 0.01% of sequences were incorrectly annotated at or above the level of the class, 0.05% were incorrectly annotated at or above the level of order, and 0.17% to 0.95% were incorrectly annotated at or above the level of family. Error rates rose only at the level of genus and above, with the total for all genes ranging from 0.73% (minimum estimate) to 3.47% (maximum estimate). For *CO1* and *Cytb*, the minimum and maximum estimates ranged from 0.58% to 3.30% and from 0.72% to 5.03%, respectively.

**Table 1.** Estimated numbers of metazoan mitochondrial encoded sequences in GenBank with incorrect taxonomic labels at the phylum, class, order, family, and genus levels

Level	No. of mislabeled sequences		% of mislabeled sequences	
	Minimum	Maximum	Minimum	Maximum
Phylum	375	375	0.01	0.01
Class	537	566	0.01	0.01
Order	1,610	2,377	0.04	0.05
Family	7,500	41,677	0.17	0.95
Family*	5,919	34,071	0.14	0.78
Genus	32,062	152,157	0.73	3.47
Genus*	29,198	140,212	0.67	3.22

Proportions are calculated based on the total number of sequences in nonsolitary clusters at the 97% clustering threshold.

* Removing sequences of Porifera and Cnidaria (0.6% and 0.09% of sequences, respectively).

**Fig. 3.**
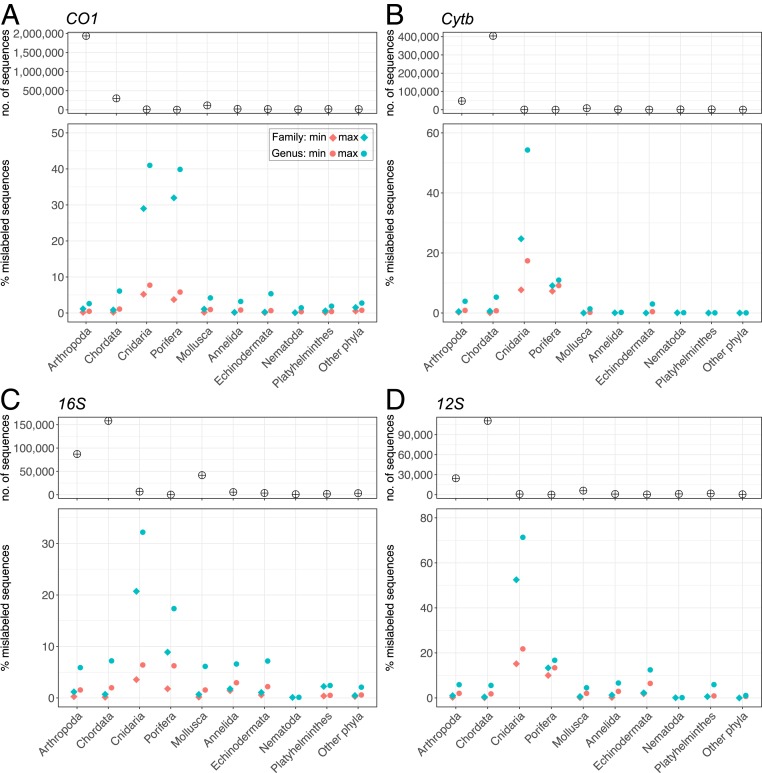
Number of sequences and estimated percentage of mislabeled sequences at the genus and family levels across major metazoan phyla: (*A*) *CO1*; (*B*) *Cytb*; (*C*) *16S*; and (*D*) *12S*. The category “Other phyla” includes sequences of Acanthocephala, Brachiopoda, Bryozoa, Chaetognatha, Ctenophora, Cycliophora, Entoprocta, Gastrotricha, Hemichordata, Kinorhyncha, Nematomorpha, Nemertea, Onychophora, Placozoa, Priapulida, Rhombozoa, Rotifera, Tardigrada, and Xenacoelomorpha.

A maximum estimate is likely to be an overestimate because it assumes that the taxon with the fewest sequences in a cluster is the one that is correctly identified (*SI Appendix*), whereas the correct annotation was the most common taxon in 95% of the clusters examined (*SI Appendix*, Fig. S3). Other limitations inherent to distance-based clustering approaches with fixed thresholds may also inflate our estimates. Moreover, some groups, such as cichlid fishes, elephants, dolphins, and bovines, as well as the higher-level taxa Anthozoa and Porifera (Fig. 3), are known to have genera that cannot be differentiated using mitochondrial gene sequences alone due to recent divergences (29) or slow rates of molecular evolution (30, 31) and are not misannotations. Our analysis shows remarkably low rates of mislabeling at the genus level within the hyperdiverse Arthropoda (0.44% to 2.56%) for the animal DNA barcode *CO1* gene (Fig. 3 and *SI Appendix*, Fig. S4), which may reflect high standards of data curation, notably in BOLD (32). Our analysis excluded the small proportion of sequences (i.e., ∼5% of *CO1* sequences and <10% of the sequences for other genes; Fig. 1 and *SI Appendix*, Fig. S1) that did not cluster with other sequences (i.e., singletons) and likely belong to rare and undersampled taxa. Although we have no a priori reason to expect a higher proportion of mislabeled sequences among singletons than within the fraction of the database analyzed, complementary analyses are warranted. The difficulty of assessing the accuracy of singleton sequences also highlights the importance of replication within taxa, particularly in barcoding initiatives, to allow cross-validation.

To determine the likely sources of taxonomic errors and hence potential remedies, we individually examined all multitaxon clusters that contained multiple phyla, classes, or orders (*SI Appendix*, Table S2 and Fig. S5). For 70.8% of the cases, there was no obvious explanation. For the remainder, the likely sources of error were laboratory contaminants (16.3%), data entry errors (8.4%, potentially due to autofill functions of sequence submission platforms), contamination by associated organisms in the sample (3.2%), and pseudogenes (1.1%) (*SI Appendix*, Table S2). As has been noted previously (33), many of these errors could be detected before submission by performing a BLAST search. For known pseudogenes, errors occur when the organelle is specified as “mitochondrion” because the sequence is most likely in the nuclear genome.

We also examined sources of error at lower taxonomic levels (families and genera), focusing on clusters containing 100 or more sequences. Interestingly, 55.0% of the sequences had the incorrect label because of taxonomic revisions, meaning that they were correct at the time of entry. Other assignment errors at these lower levels were caused by laboratory contamination (4.5%) and data entry errors (2.8%). The more numerous examples of misannotations at the family or genus level in small clusters that we did not examine are less likely due to data entry and contamination errors, because they would normally lead to misidentifications at higher taxonomic levels; for these, there is no substitute for having appropriate taxonomic expertise when assigning names to samples. As has been noted by others, taxonomic names for submitted sequences should not be based solely on a database search, to prevent the propagation of errors (34).

Concerns about errors in GenBank have prompted cautionary notes (35–37) and the creation of curated sequence databases for particular taxonomic groups and genes, including BOLD (32), PR2 (38), SILVA (39), PHYMYCO-DB (40), and MIDORI (41). However, our results stand in contrast to most previous estimates of GenBank annotation error rates. For example, Bridge et al. (42) reported that 12 of 51 species of the renowned, highly poisonous fungus *Amanita* had incorrect genus names and that 16 of 100 fungi of the order Helotiales were misidentified at the genus level; in most of these cases, the annotations were to distantly related fungi or nonfungi. A recent study estimated that 6.9% of all sequences listed as Gastrotricha (43) did not belong to this phylum (our estimate is 2.5%). Similarly, some researchers have warned that the number of misidentified parasite sequences may be increasing (26), although there are no quantitative estimates. There are several possible reasons for the discrepancy between earlier estimates and concerns and our results. Specifically, fungi, parasites, and other small metazoans are more taxonomically challenging and more difficult to sample without contamination. Error rates may also have decreased in recent years, with increasing attention to quality control (e.g., GenBank; https://www.ncbi.nlm.nih.gov/books/NBK44940/).

**Fig. 4.**
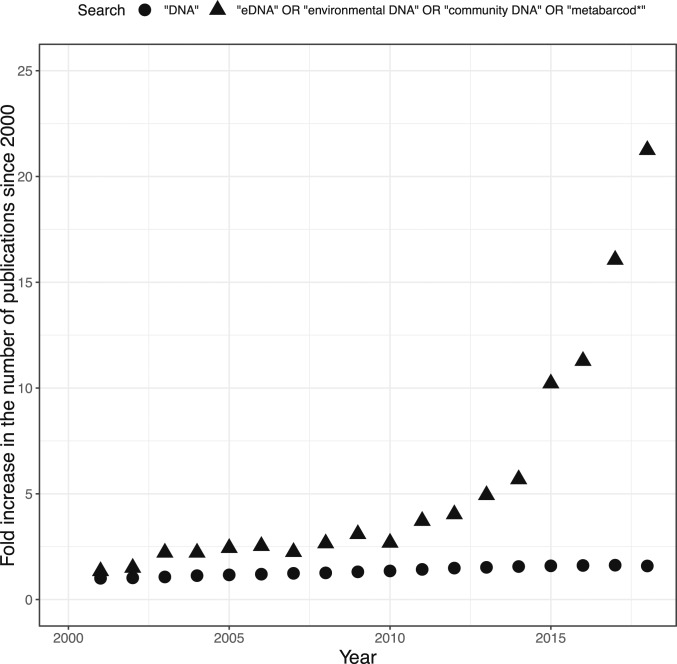
Increase in the number of scientific publications since 2000 based on Web of Science using the terms “eDNA” or “environmental DNA” or “community DNA” or “metabarcod*” compared with the comparatively stable number of publications using the term term “DNA”.

DNA-based biosurveys are rapidly increasing in popularity (4, 44), with the annual number of papers based on these methods having risen >21-fold between 2000 and 2018 (Fig. 4). Our results suggest that while quality control efforts remain essential, metazoan sequences in GenBank are highly reliable for a range of applications, ranging from underpinning ecological inferences to assessing environmental policies.

## Materials and Methods

The sequence datasets for 13 protein-coding mitochondrial genes—ATP synthase subunit 6 (*A6*) and 8 (*A8*); Cytochrome oxidase subunits I (*CO1*), II (*CO2*), and III (*CO3*); Cytochrome *b* apoenzyme (*Cytb*); NADH dehydrogenase subunits 1 to 4 (*ND1* to *ND4*), 4 L (*ND4L*), 5 (*ND5*), and 6 (*ND6*)—and 2 ribosomal RNA-coding mitochondrial genes—large (*lrRNA*) and small (*srRNA*) ribosomal subunit RNA—were prepared using BLAST with reference datasets prepared from RefSeq mitochondrial genome datasets (downloaded on Feb 21, 2018) to perform the gene classification as described below (flowchart in *SI Appendix*, Fig. S6). (Because various gene names have been used to specify a single gene in GenBank, one cannot simply use the annotated gene names to classify the sequences to the genes.)

First, the BLAST NT FASTA file was downloaded from the National Center for Biotechnology Information server (ftp://ftp.ncbi.nih.gov/blast/db/FASTA) on February 21, 2018. Next, mitochondria-related gene sequences were extracted from the FASTA file. Then GenBank flat files of the mitochondria-related gene sequences were further downloaded using NCBI EDirect. Next, only the metazoan flat files were extracted from the flat files. From the flat files, each gene sequence was truncated using gene location information, and separate FASTA files were prepared for each gene. Taxonomic names (phylum, class, order, family, genus, and species) were added to each sequence using taxonomy files (nodes.dmp and names.dmp) downloaded from ftp://ftp.ncbi.nih.gov/pub/taxonomy. For the 13 protein-coding genes, corresponding amino acid FASTA files were prepared, since amino acid sequences were more efficient than nucleotide sequences for separating the genes via BLAST classification. These prepared FASTA files (nucleotide sequences for ribosomal RNA-coding genes and amino acid sequences for protein-coding genes) were used as queries for BLAST.

The following BLAST parameters were used for protein-coding genes: blastp -db RefSeq_prepared_db -query metazoan_mitochondrial_amino_acid.fasta -num_alignments 100 -word_size 3 -outfmt 7 -seg ‘no’ -soft_masking ‘false’. The following BLAST parameters were used for ribosomal RNA-coding genes: blastn -db RefSeq_prepared_db -query metazoan_mitochondrial_nucleotide.fasta -num_alignments 100 -word_size 11 -outfmt 7 -dust ‘no’ -soft_masking ‘false’. BLAST results were considered significant only if 2 criteria were met: E-value <1e^−10^ and bit score >70. If multiple genes were listed in the BLAST result, the queries were discarded.

Based on the BLAST result, we separated the dataset by genes (total of 15). In addition, length restriction was performed for the datasets: *12S*, 200 to 2,000; *16S*, 100 to 2,500; *A6*, 100 to 1,000; *A8*, 100 to 500; *CO1*, 100 to 2,000; *CO*_*2*_, 100 to 1,500; *CO3*, 100 to 1,300; *Cytb*, 100 to 1,500; *ND1*, 50 to 1,200; *ND2*, 150 to 1,500; *ND3*, 100 to 600; *ND4*, 150 to 2,000; *ND4L*, 100 to 700; *ND5*, 150 to 2,000; *ND6*, 150 to 1,500. This length restriction procedure was required to avoid random clustering of short sequences in the subsequent procedure.

To identify taxonomically mislabeled sequences, clustering analyses were performed at 97%, 98%, 99%, and 100% similarity thresholds with VSEARCH (27) (–sortbylength,–cluster_fast). The vast majority of sequences were located in multisequence clusters at 97% for all genes (from 86.2% for *12S* to 95.6% for *CO1*; *SI Appendix*, Fig. S1). After clustering, we checked incongruences of taxonomic labels within each cluster at all taxonomic levels except species (i.e., phylum, class, order, family, and genus) using custom Perl scripts. Clusters with sequences labeled as different phyla, classes, orders, families, or genera were extracted for downstream analysis.

We attempted to identify which were the mislabeled sequences in all clusters containing multiple phyla, classes, and orders, but we examined only a portion of the much more numerous clusters with multiple families and genera. We focused on clusters containing more than 100 sequences, because our estimates based on order-level misidentifications showed that manually checking larger clusters was a relatively efficient way to narrow the possible range of error rates. Clusters with more than 100 sequences represented only 2.0% of all clusters but contained 46.8% of the total sequences. By simulating sampling the order-level data at different depths, we found that randomly selecting sequences and then checking the corresponding clusters was inappropriate, because this method tended to preferentially sample low-error large clusters, resulting in a poor approximation of the true error rate (*SI Appendix*, Fig. S2). While randomly sampling clusters directly produced a more accurate estimate of the error rate, we opted to manually check large clusters by hand and provide a range of possible error rates for family and genus misidentifications so as to err on the conservative side and avoid the need to specify a model.

A similarity search using the BLAST server (33) (blastn with “low-complexity region filter” and “mask for lookup table only” functions disabled) was performed using the putative mislabeled sequence as a query, and the distance tree function on the BLAST server was used to examine phylogenetic relationships with 100 close matches to the query. If the taxonomy of the outgroup sequences disagreed with the query taxonomy, we concluded that the query had the wrong taxonomy. Mislabeled sequences at the phylum, class, and order levels detected in this study will be reported to GenBank for removal from BLAST search databases (i.e., flat files flagged as “UNVERIFIED”).

Mislabeled sequences could not be identified unequivocally in all clusters at the phylum, class, and order levels or in large clusters (>100 sequences) with multiple families and genera (incorrect taxon label identified in 65% of clusters examined). Moreover, we did not attempt to identify which sequences were mislabeled in the numerous small clusters (<100 sequences) containing multiple families or genera. In all these cases, we estimated the minimum and maximum numbers of sequences that were possibly mislabeled in each of these clusters as follows. To calculate the maximum number of mislabeled sequences, we selected the taxon within a cluster that had the smallest number of entries and assumed that all the remaining sequences in the cluster were in error. To calculate the minimum number of mislabeled sequences, we selected the taxon with the largest number of entries and assumed that all the remaining sequences were in error. For example, if a cluster of 6 sequences contained 3 sequences labeled taxon A, 2 sequences labeled taxon B, and 1 sequence labeled taxon C, then the minimum number of mislabeled sequences would be 3 and the maximum number would be 5.

The putative cause of errors of each sequence unambiguously identified as mislabeled was inferred to the extent possible by using detailed observations of the taxonomic affiliation and sequence annotations within each cluster (identification flowchart in *SI Appendix*, Fig. S5). First, we classified sequences as mislabeled because of “data entry error” if the accession number and genus name had consecutive or nearly consecutive numbers or if had a very similar genus name. Next, we identified whether they were common laboratory contaminants, such as DNA from humans, common rodents, laboratory model organisms, common human food, mosquitos, or pets. We then checked whether the sequence was a potential nuclear mitochondrial pseudogene using 2 criteria: a mention of pseudogenes in the definition or project title of the GenBank flat file and a drastic change in sequence similarity between different regions of the sequence. Then the sequence was classified as a contamination from a host or dietary item if the project title of the GenBank flat file mentioned gut content analysis or parasite study. Next, we checked whether the BLAST result indicated high similarity to bacterial sequences. Finally, we searched online databases (e.g., FishBase, WORMS) to check whether a taxonomic revision took place in the group. If none of these categories applied, we classified the sequence as other laboratory contamination or taxonomic misidentification. We did not attempt to identify the cause of sequence misidentification in phyla Porifera and Cnidaria clusters because of the lower taxonomic resolution in these groups for mitochondrial genes (30, 31).

Raw data files (FASTA files: https://figshare.com/articles/Fasta_files/7642202), data analysis scripts (https://figshare.com/articles/R_Script_for_estimation_of_minimum_and_maximum_error_/7856180), clustering output files (https://figshare.com/articles/MultipleTaxaClustersFamiliyGenus/7853561; https://figshare.com/articles/MultipleTaxaClusteringPhylumClassOrder/7853567), and a table summarizing the estimated minimum and maximum numbers of mislabeled sequences per phylum and per gene (https://figshare.com/articles/Summarized_Table/7856156) are available at Figshare (https://figshare.com/).

## Supplementary Material

Supplementary File

## References

[1] Bellard C., Bertelsmeier C., Leadley P., Thuiller W., Courchamp F., Impacts of climate change on the future of biodiversity. Ecol. Lett. 15, 365–377 (2012). 22257223 10.1111/j.1461-0248.2011.01736.x PMC3880584

[2] Bohmann K., Environmental DNA for wildlife biology and biodiversity monitoring. Trends Ecol. Evol. 29, 358–367 (2014). 24821515 10.1016/j.tree.2014.04.003

[3] Hebert P. D. N., Cywinska A., Ball S. L., deWaard J. R., Biological identifications through DNA barcodes. Proc Biol Sci 270, 313–321 (2003). 12614582 10.1098/rspb.2002.2218 PMC1691236

[4] Creer S., The ecologist’s field guide to sequence-based identification of biodiversity. Methods Ecol. Evol. 7, 1008–1018 (2016).

[5] Adamowicz S. J., Trends in DNA barcoding and metabarcoding. Genome 62, v–viii (2019). 30998119 10.1139/gen-2019-0054

[6] Zinger L., Body size determines soil community assembly in a tropical forest. Mol. Ecol. 28, 528–543 (2019). 30375061 10.1111/mec.14919

[7] Lallias D., Environmental metabarcoding reveals heterogeneous drivers of microbial eukaryote diversity in contrasting estuarine ecosystems. ISME J. 9, 1208–1221 (2015). 25423027 10.1038/ismej.2014.213 PMC4409164

[8] Barsoum N., Bruce C., Forster J., Ji Y.-Q., Yu D. W., The devil is in the detail: Metabarcoding of arthropods provides a sensitive measure of biodiversity response to forest stand composition compared with surrogate measures of biodiversity. Ecol. Indic. 101, 313–323 (2019).

[9] Siegenthaler A., Wangensteen O. S., Benvenuto C., Campos J., Mariani S., DNA metabarcoding unveils multiscale trophic variation in a widespread coastal opportunist. Mol. Ecol. 28, 232–249 (2019). 30276912 10.1111/mec.14886 PMC7380037

[10] Deiner K., Environmental DNA metabarcoding: Transforming how we survey animal and plant communities. Mol. Ecol. 26, 5872–5895 (2017). 28921802 10.1111/mec.14350

[11] Del Carmen Gomez Cabrera M., Broadening the taxonomic scope of coral reef palaeoecological studies using ancient DNA. Mol. Ecol. 28, 2636–2652 (2019). 30723959 10.1111/mec.15038

[12] Epp L. S., A global perspective for biodiversity history with ancient environmental DNA. Mol. Ecol. 28, 2456–2458 (2019). 31216119 10.1111/mec.15118

[13] Benson D. A., GenBank. Nucleic Acids Res. 41, D36–D42 (2013). 23193287 10.1093/nar/gks1195 PMC3531190

[14] Strasser B. J., GenBank—Natural history in the 21st century? Science 322, 537–538 (2008). 18948528 10.1126/science.1163399

[15] Porter T. M., Hajibabaei M., Over 2.5 million COI sequences in GenBank and growing. PLoS One 13, e0200177 (2018). 30192752 10.1371/journal.pone.0200177 PMC6128447

[16] Elbrecht V., Vamos E. E., Meissner K., Aroviita J., Leese F., Assessing strengths and weaknesses of DNA metabarcoding-based macroinvertebrate identification for routine stream monitoring. Methods Ecol. Evol. 8, 1265–1275 (2017).

[17] O’Donnell J. L., Kelly R. P., Lowell N. C., Port J. A., Indexed PCR primers induce template-specific bias in large-scale DNA sequencing studies. PLoS One 11, e0148698 (2016). 26950069 10.1371/journal.pone.0148698 PMC4780811

[18] Hansen B. K., Bekkevold D., Clausen L. W., Nielsen E. E., The sceptical optimist: Challenges and perspectives for the application of environmental DNA in marine fisheries. Fish Fish. 19, 751–768 (2018).

[19] Havird J. C., Sloan D. B., The roles of mutation, selection, and expression in determining relative rates of evolution in mitochondrial versus nuclear genomes. Mol. Biol. Evol. 33, 3042–3053 (2016). 27563053 10.1093/molbev/msw185 PMC5100045

[20] Boore J. L., Animal mitochondrial genomes. Nucleic Acids Res. 27, 1767–1780 (1999). 10101183 10.1093/nar/27.8.1767 PMC148383

[21] Mora C., Tittensor D. P., Adl S., Simpson A. G. B., Worm B., How many species are there on Earth and in the ocean? PLoS Biol. 9, e1001127 (2011). 21886479 10.1371/journal.pbio.1001127 PMC3160336

[22] Kryukov K., Imanishi T., Human contamination in public genome assemblies. PLoS One 11, e0162424 (2016). 27611326 10.1371/journal.pone.0162424 PMC5017631

[23] Siddall M. E., Fontanella F. M., Watson S. C., Kvist S., Erséus C., Barcoding bamboozled by bacteria: Convergence to metazoan mitochondrial primer targets by marine microbes. Syst. Biol. 58, 445–451 (2009). 20525598 10.1093/sysbio/syp033

[24] Strong M. J., Microbial contamination in next-generation sequencing: Implications for sequence-based analysis of clinical samples. PLoS Pathog. 10, e1004437 (2014). 25412476 10.1371/journal.ppat.1004437 PMC4239086

[25] Bensasson D., Zhang D., Hartl D. L., Hewitt G. M., Mitochondrial pseudogenes: Evolution’s misplaced witnesses. Trends Ecol. Evol. 16, 314–321 (2001). 11369110 10.1016/s0169-5347(01)02151-6

[26] Valkiūnas G., Atkinson C. T., Bensch S., Sehgal R. N. M., Ricklefs R. E., Parasite misidentifications in GenBank: How to minimize their number? Trends Parasitol. 24, 247–248 (2008). 18440865 10.1016/j.pt.2008.03.004

[27] Rognes T., Flouri T., Nichols B., Quince C., Mahé F., VSEARCH: A versatile open source tool for metagenomics. PeerJ 4, e2584 (2016). 27781170 10.7717/peerj.2584 PMC5075697

[28] Lessios H. A., The great American schism: Divergence of marine organisms after the rise of the Central American isthmus. Annu. Rev. Ecol. Evol. Syst. 39, 63–91 (2008).

[29] Hickerson M. J., Meyer C. P., Moritz C., DNA barcoding will often fail to discover new animal species over broad parameter space. Syst. Biol. 55, 729–739 (2006). 17060195 10.1080/10635150600969898

[30] Huang D., Meier R., Todd P. A., Chou L. M., Slow mitochondrial COI sequence evolution at the base of the metazoan tree and its implications for DNA barcoding. J. Mol. Evol. 66, 167–174 (2008). 18259800 10.1007/s00239-008-9069-5

[31] Shearer T. L., Van Oppen M. J. H., Romano S. L., Wörheide G., Slow mitochondrial DNA sequence evolution in the Anthozoa (Cnidaria). Mol. Ecol. 11, 2475–2487 (2002). 12453233 10.1046/j.1365-294x.2002.01652.x

[32] Ratnasingham S., Hebert P. D. N., Bold: The barcode of life data system (http://www.barcodinglife.org). *Mol. Ecol. Notes* 7, 355–364 (2007). 10.1111/j.1471-8286.2007.01678.x PMC1890991 18784790

[33] Altschul S. F., Gapped BLAST and PSI-BLAST: A new generation of protein database search programs. Nucleic Acids Res. 25, 3389–3402 (1997). 9254694 10.1093/nar/25.17.3389 PMC146917

[34] Belcaid M., Poisson G., Detecting anomalies in the Cytochrome C Oxidase I amplicon sequences using minimum scoring segments. Appl. Comput. Rev. 17, 6–14 (2018).

[35] Bidartondo M. I., Preserving accuracy in GenBank. Science 319, 1616 (2008). 10.1126/science.319.5870.1616a 18356505

[36] Harris D. J., Can you bank on GenBank? Trends Ecol. Evol. 18, 317–319 (2003).

[37] Nilsson R. H., Taxonomic reliability of DNA sequences in public sequence databases: A fungal perspective. PLoS One 1, e59 (2006). 17183689 10.1371/journal.pone.0000059 PMC1762357

[38] Guillou L., The protist ribosomal reference database (PR2): A catalog of unicellular eukaryote small sub-unit rRNA sequences with curated taxonomy. Nucleic Acids Res. 41, D597–D604 (2013). 23193267 10.1093/nar/gks1160 PMC3531120

[39] Quast C., The SILVA ribosomal RNA gene database project: Improved data processing and web-based tools. Nucleic Acids Res. 41, D590–D596 (2013). 23193283 10.1093/nar/gks1219 PMC3531112

[40] Mahé S., PHYMYCO-DB: A curated database for analyses of fungal diversity and evolution. PLoS One 7, e43117 (2012). 23028445 10.1371/journal.pone.0043117 PMC3441585

[41] Machida R. J., Leray M., Ho S.-L., Knowlton N., Metazoan mitochondrial gene sequence reference datasets for taxonomic assignment of environmental samples. Sci. Data 4, 170027 (2017). 28291235 10.1038/sdata.2017.27 PMC5349245

[42] Bridge P. D., Roberts P. J., Spooner B. M., Panchal G., On the unreliability of published DNA sequences. New Phytol. 160, 43–48 (2003). 10.1046/j.1469-8137.2003.00861.x 33873520

[43] Mioduchowska M., Czyż M. J., Gołdyn B., Kur J., Sell J., Instances of erroneous DNA barcoding of metazoan invertebrates: Are universal cox1 gene primers too “universal”? PLoS One 13, e0199609 (2018). 29933389 10.1371/journal.pone.0199609 PMC6014667

[44] Leray M., Knowlton N., Censusing marine eukaryotic diversity in the twenty-first century. Philos. Trans. R. Soc. Lond. B Biol. Sci. 371, 20150331 (2016). 27481783 10.1098/rstb.2015.0331 PMC4971183

