# Supplementary material for: GenBank is a reliable resource for 21st century biodiversity research
Source: Proc Natl Acad Sci U S A. 2019 Oct 21;116(45):22651–6. doi: 10.1073/pnas.1911714116 (PMC6842603; doi:10.1073/pnas.1911714116)
Supplement: Supplementary File [file pnas.1911714116.sapp.pdf]

PNAS
www.pnas.org

Supplementary Information for

GenBank is a reliable resource for 21st century biodiversity research

Matthieu Leray
Nancy Knowlton
Shian-Lei Ho
Bryan N. Nguyen
Ryuji J. Machida

Corresponding Authors
Ryuji J. Machida; Biodiversity Research Centre, Academia Sinica, Taipei, 115-29, Taiwan; ryujimachida@gmail.com; Tel +886-2-2787-1585; Fax +886-2-2785-8059
Nancy Knowlton; National Museum of Natural History, Smithsonian, Washington, DC, USA; knowlton@si.edu; Tel +1-202-213 4587

This PDF file includes:

Figs. S1 to S6
Tables S1 to S2

www.pnas.org/cgi/doi/10.1073/pnas.1911714116

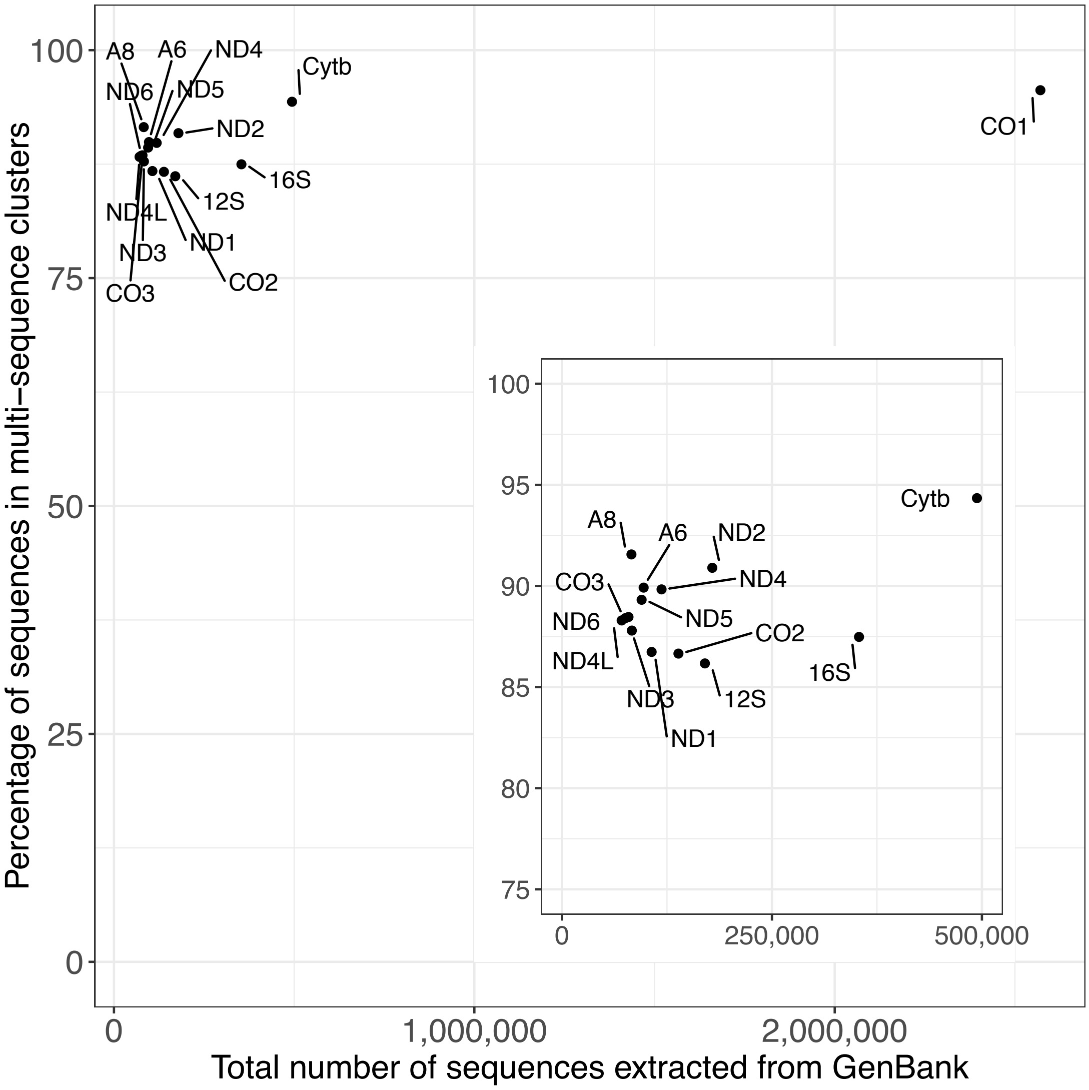

Fig. S1. Proportion of metazoan sequences that clustered at 97% similarity threshold as a function of the total number of sequences present in the GenBank BLAST nucleotide database.

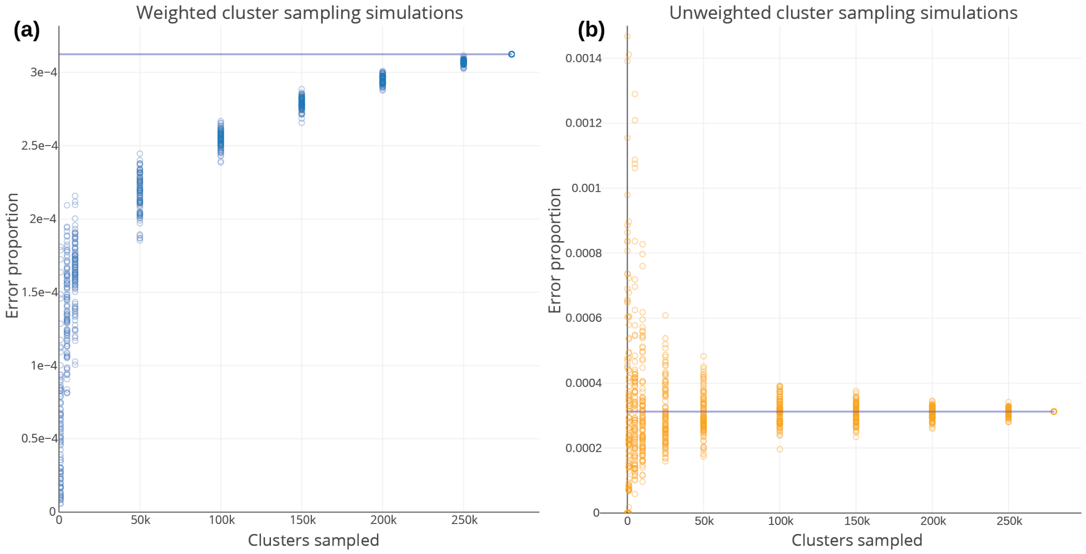

Fig. S2. Order-level error proportion estimates resulting from random weighted (a) and unweighted (b) subsampling of the data for 100 replicates at increasing sampling depths. The horizontal blue line represents the total order-level error proportion of the entire dataset. Subsampling clusters, weighting by cluster size, is equivalent to randomly sampling sequences for their corresponding clusters.

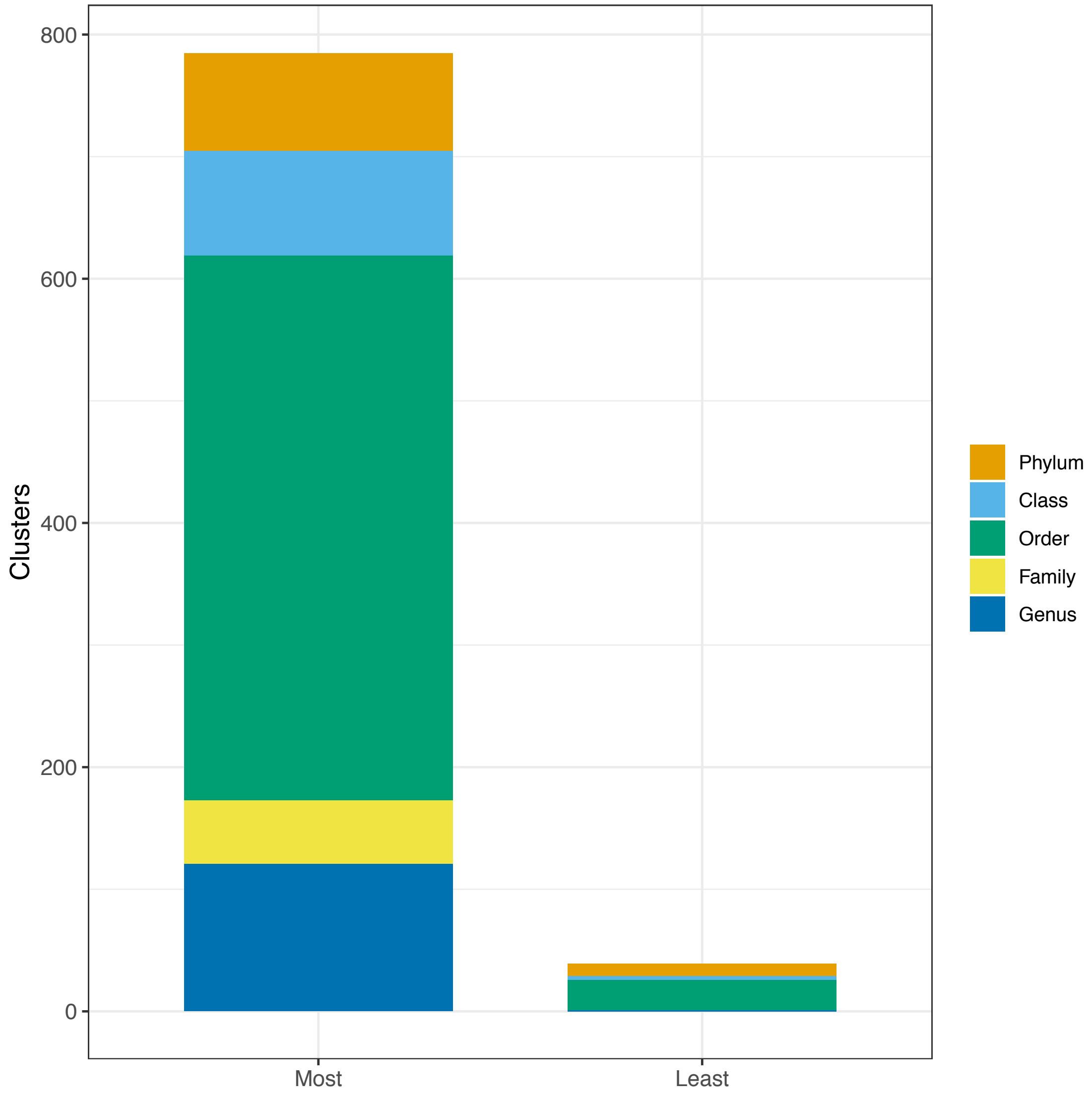

Fig. S3. Number of clusters for which the most or least common entries had correct taxonomic annotations. Clusters with equal number of entries belonging to the most and least common sequences were disregarded in this estimation.

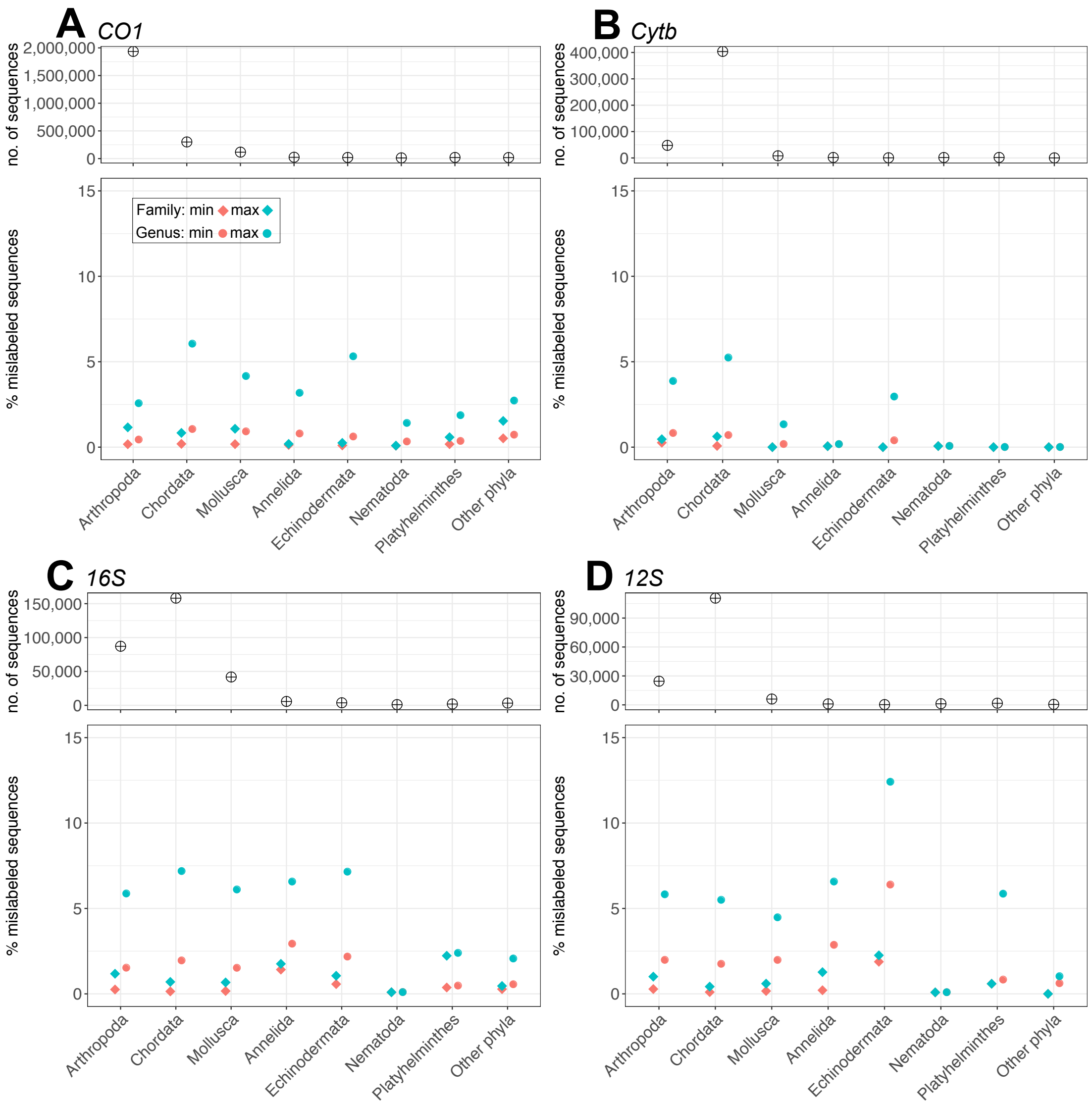

Fig. S4. Estimated percentage of mislabeled sequences at the genus and family levels for *CO1* (A), *Cytb* (B), *16S* (C) and *12S* (D) across major metazoan phyla. We excluded phyla Porifera and Cnidaria clusters with their higher error estimates likely caused by slower rates of molecular evolution to facilitate visualization of lower error rates in the other groups. The category "Other phyla" includes sequences of Acanthocephala, Brachiopoda, Bryozoa, Chaetognatha, Ctenophora, Cycliophora, Entoprocta, Gastrotricha, Hemichordata, Kinorhyncha, Nematomorpha, Nemertea, Onychophora, Placozoa, Priapulida, Rhombozoa, Rotifera, Tardigrada and Xenacoelomorpha.

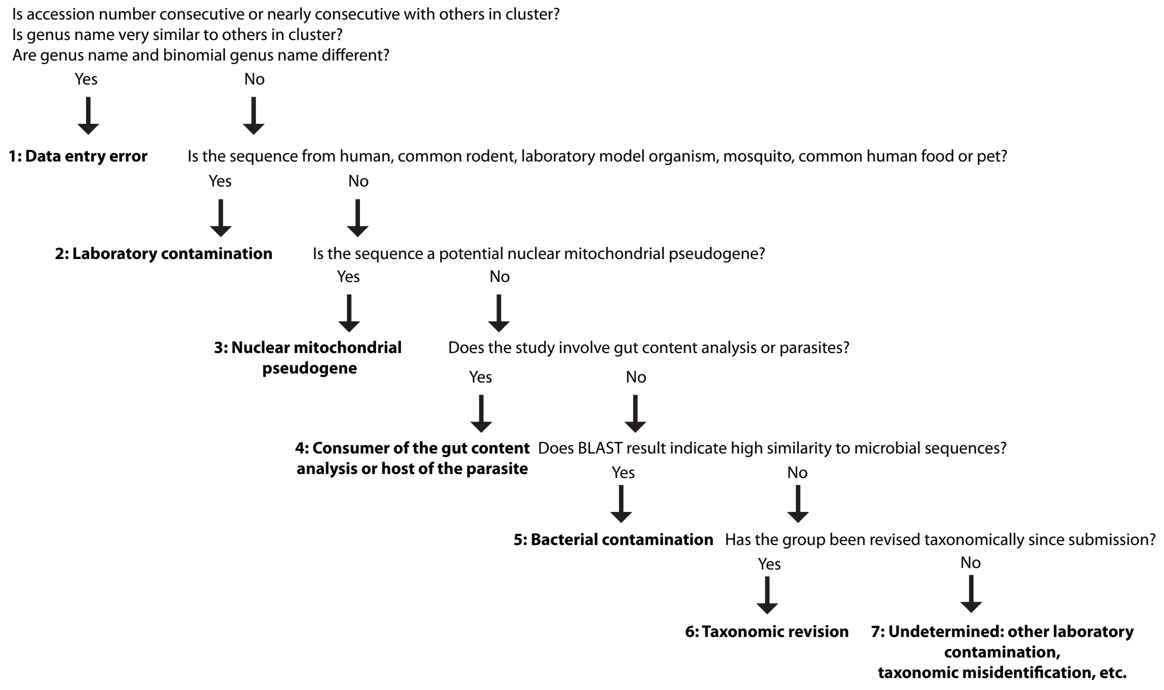

Fig. S5. Flowchart for determining causes of multiple taxa (genus, family, order, class, phylum) within clusters. For each potentially mislabeled sequence the questions shown were answered in sequence to determine the most likely reason.

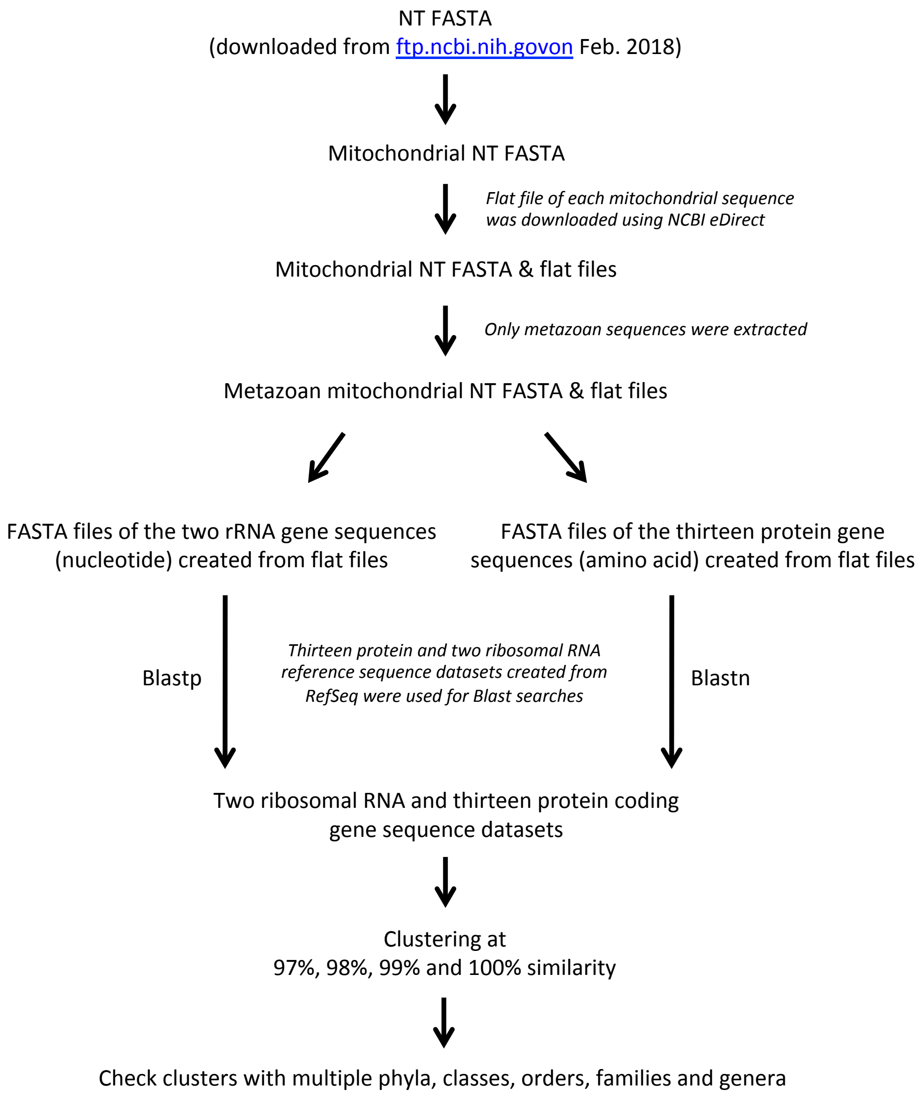

Fig. S6. Flowchart describing the procedure used to classify mitochondrial gene sequences downloaded from GenBank.

	Total number of clusters	Number of non-solitary clusters	Clusters with multiple phyla		Clusters with multiple classes		Clusters with multiple orders		Clusters with multiple families		Clusters with multiple genera		Total clusters with multiple taxa	
Genes	Count	Count	Count	%	Count	%	Count	%	Count	%	Count	%	Count	%
12S	38287	14760	1	0.01	2	0.01	49	0.33	132	0.89	1011	6.85	1195	8.10
16S	77890	33615	16	0.05	10	0.03	80	0.24	354	1.05	2059	6.13	2519	7.49
A6	13895	4098	0	0.00	0	0.00	13	0.32	20	0.49	170	4.15	203	4.95
A8	10047	3070	0	0.00	0	0.00	10	0.33	22	0.72	204	6.64	236	7.69
Cytb	56805	28854	18	0.06	16	0.06	78	0.27	145	0.50	919	3.19	1176	4.08
CO1	261345	148475	71	0.05	78	0.05	381	0.26	1409	0.95	3852	2.59	5791	3.90
CO2	26846	8345	1	0.01	2	0.02	12	0.14	51	0.61	383	4.59	449	5.38
CO3	11908	2778	0	0.00	0	0.00	10	0.36	35	1.26	147	5.29	192	6.91
ND1	19714	5561	0	0.00	1	0.02	7	0.13	34	0.61	192	3.45	234	4.21
ND2	28331	12045	0	0.00	1	0.01	14	0.12	43	0.36	328	2.72	386	3.20
ND3	13754	3608	0	0.00	0	0.00	7	0.19	25	0.69	158	4.38	190	5.27
ND4	18261	6209	1	0.02	0	0.00	7	0.11	24	0.39	189	3.04	221	3.56
ND4L	10660	2348	0	0.00	0	0.00	8	0.34	29	1.24	152	6.47	189	8.05
ND5	13814	3688	0	0.00	0	0.00	5	0.14	19	0.52	178	4.83	202	5.48
ND6	11176	2445	0	0.00	1	0.04	7	0.29	22	0.90	132	5.40	162	6.63
**Total**	**612733**	**279899**	**108**		**111**		**688**		**2364**		**10074**		**13345**	

Table S1. Observed number of clusters, non-solitary clusters, clusters with multiple phyla, classes, orders, families, and genera at 97% clustering. Percentages are calculated relative to the total number of non-solitary clusters. Clusters with multiple taxonomic groups were independently counted at each taxonomic level (i.e. multiple class counts were those in addition to those occurring via multiple phyla counts).

		12S	*16S*	*A6*	*A8*	*Cytb*	*CO1*	*CO2*	*CO3*	*ND1*	*ND2*	*ND3*	*ND4*	*ND4L*	*ND5*	*ND6*	**Total no. seqs**	**% seqs**
Phylum, class and order levels	1: Data entry error	8	6	0	0	16	88	1	0	1	0	0	0	0	1	0	121	8.4
	2: Laboratory cont.	6	95	1	0	45	75	11	1	0	0	0	0	0	1	0	235	16.3
	3: Pseudogene	4	1	3	3	2	2	0	0	0	0	0	0	1	0	0	16	1.1
	4: Consumer or host	0	3	0	0	42	1	0	0	0	0	0	0	0	0	0	46	3.2
	5: Bacterial cont.	0	0	0	0	0	2	0	0	0	0	0	0	0	0	0	2	0.1
	6: Taxonomic revision	0	0	0	0	0	0	0	0	0	0	0	0	0	0	0	0	0.0
	7: Undetermined	44	103	9	55	124	622	16	8	3	10	5	10	3	2	5	1019	70.8
	Total	62	208	13	58	229	790	28	9	4	10	5	10	4	4	5	1439	100

		12S	*16S*	*A6*	*A8*	*Cytb*	*CO1*	*CO2*	*CO3*	*ND1*	*ND2*	*ND3*	*ND4*	*ND4L*	*ND5*	*ND6*	**Total no. seqs**	**% seqs**
Family and genus levels	1: Data entry error	2	0	0	0	4	16	0	0	0	0	0	0	0	0	0	22	2.8
	2: Laboratory cont.	2	1	0	0	20	10	0	0	0	0	0	1	0	1	0	35	4.5
	3: Pseudogene	0	0	0	0	0	0	1	0	0	0	0	0	0	0	0	1	0.1
	4: Consumer or host	0	0	0	0	0	0	0	0	0	0	0	0	0	0	0	0	0.0
	5: Bacterial cont.	0	0	0	0	0	0	0	0	0	0	0	0	0	0	0	0	0.0
	6: Taxonomic revision	0	0	0	0	421	9	1	0	0	0	0	0	0	0	0	431	55.0
	7: Undetermined	2	15	1	0	72	189	7	0	0	3	2	0	0	3	0	294	37.5
	Total	6	16	1	0	517	224	9	0	0	3	2	1	0	4	0	783	100

Table S2. Potential causes of sequence mislabeling at the phylum, class, order, family and genus levels for metazoan sequences belonging to 15 mitochondrial encoded genes. Only clusters where mislabeled sequences could be unequivocally identified were examined. Moreover, we only examined clusters with multiple families and genera when they contained at least 100 sequences. (cont. = contamination)
